# A Multiscale Simulation of Polymer Melt Injection Molding Filling Flow Using SPH Method with Slip-Link Model

**DOI:** 10.3390/polym14204334

**Published:** 2022-10-14

**Authors:** Mengke Ren, Junfeng Gu, Zheng Li, Shilun Ruan, Changyu Shen

**Affiliations:** 1 State Key Laboratory of Structural Analysis for Industrial Equipment, Department of Engineering Mechanics, Dalian University of Technology, Dalian 116024, China; 2 Zhengzhou College of Dalian University of Technology, Zhengzhou 450016, China; 3 School of Materials Science and Engineering, The Key Laboratory of Material Processing and Mold of Ministry of Education, Zhengzhou University, Zhengzhou 450002, China

**Keywords:** multiscale simulation, injection molding, smoothed particle hydrodynamics, slip-link model

## Abstract

In this article, a multiscale simulation method of polymer melt injection molding filling flow is established by combining an improved smoothed particle hydrodynamics method and clustered fixed slip-link model. The proposed method is first applied to the simulation of HDPE melt in a classic Poiseuille flow case, and then two high-speed and high-viscosity injection molding flow cases in two simple long 2D rectangular cavities with and without a circular obstacle, respectively, are analyzed. For each case, the macro velocity results, and the micro average number of entanglements *Z*_ave_ and orientation degree *S* results are demonstrated and discussed, and the changing trends of *Z*_ave_ and *S* are analyzed. The results of the two injection molding cases are compared, and the influence of the obstacle on the injection flow at both the macro and micro levels is analyzed. Furthermore, based on the multiscale results, reason of some structural features and defects in injection molded products are analyzed.

## 1. Introduction

The injection molding process is a polymer molding processing method with popularity and efficiency [1,2]. With the growing performance demands for injection molding products, some in-depth research on the injection molding process becomes necessary. As the macroscopic product properties and defects reflect the microscopic polymer structure, a multiscale simulation of injection molding can be implemented for an insight of the influence of macroscopic injection molding filling flow history on the evolution of the microscopic polymer chain conformation.

The macroscopic simulation of injection molding has been researched and developed for decades, and many simulation methods have been proposed. The mainstream method for injection molding filling simulation is the mesh-based approaches [3,4]. The most popular injection molding engineering software Moldflow uses the finite element method (FEM), and Modex3D uses the finite volume method (FVM). Rather than the mesh-based approaches, some meshfree methods [5,6,7,8] for macroscopic injection molding simulation have developed rapidly in recent years. As a meshfree method based on Lagrangian description, the smoothed particle hydrodynamics (SPH) method [9,10] has massive applications in the study of free-surface flow [11,12,13,14], impact and explosion [15,16,17,18,19,20], fracture [21,22], metal processing [23,24,25] and other fields, and is applied to the injection molding in recent years [26,27,28,29,30]. The SPH method comes with trace tracking of the SPH particles, which can also be regarded as material points. Hence one SPH particle can be directly coupled with a microscopic simulation ensemble, and a simpler multiscale simulation approach can be realized.

As for the microscopic simulation, there are atomic-scale methods such as molecular dynamics and its coarse-grained versions [31], as well as methods at the polymer chain scale such as tube model [32,33,34,35] and slip-link model. The slip-link model focuses on the entanglements between the polymer chains, and has a single-chain version proposed by Schieber [36], and a multi-chain version proposed by Masubuchi [37]. Although the single-chain slip-link model has rougher description of polymer chain conformation, it has lower computational cost, and in the recent coarse-grained version called clustered fixed slip-link model (CFSM) [38], it is possible to simulate polymer chains with very high molecular weights equivalent to that of polymer for injection molding.

Combining SPH method and slip-link model, some scholars have made attempts in the multiscale simulation of polymer flow. Feng et al. [39] simulated the flow past a periodic array of confined cylinders and the journal bearing lubricated with polymer melt, and the results are in good agreement with the simulation results of the UCM viscoelastic constitutive equation. Murashima et al. [40] simulated the 2D flow around two cylinders, and obtained the influence of the cylindrical obstacles on the microscopic variables, including the mean length of the polymer chains, the mean number of entanglements and degree of orientation. Sato et al. [41] simulated flows in a 4:1:4 contraction-expansion channel, evaluated the microscopic variables and developed a model equation that describes the time evolution of the number density of entanglements along a polymer chain. However, the multiscale simulations in these works are all some classical flow studies in a closed space filled with polymer melt, which is much simpler than the flow in the polymer processing such as injection molding filling. The injection molding filling flow involves complex boundary conditions such as that at melt front (free surface), along with heat conduction and phase transitions.

The main purpose of this work is to make multiscale simulation closer to engineering applications, so a new multiscale simulation strategy of polymer melt injection molding filling flow is established by combining an improved SPH method and CFSM, and applied to HDPE melt in a classic Poiseuille flow case and two injection molding cases in two simple long 2D rectangular cavities with and without a circular obstacle, respectively. The rest content of this article is arranged as follows: In Section 2, the macro governing equations and their discretization using the improved SPH algorithm, the micro CFSM and the multiscale simulation solution procedure are described. In Section 3, the multiscale simulation method is implemented in the various examples described above. For each case, the macro velocity results, and the micro average number of entanglements and orientation degree results of multiscale simulation will be demonstrated and discussed. In the two injection molding cases, the above simulation results together with the tracking line quantity results that provide an insight of melt motion of the two examples are compared. Finally, in Section 4, there are conclusions and remarks.

## 2. Formulations

### 2.1. Smoothed Particle Hydrodynamics

#### 2.1.1. Governing Equations

In this work, on the macro scale, the polymer melt is considered as an isothermal, transient, weakly compressible viscous fluid, and the basic governing equations can be written as Equations (1) and (2):

(1) $$\frac{\partial \rho }{\partial t}=-\rho (\nabla \cdot u),$$

(2) $$\rho \frac{\partial u}{\partial t}=-\nabla p+\mu \nabla^{2}u+\rho f,$$

where *ρ* is the density, ***u*** is the velocity, ***f*** is the external force acceleration, and *p* is the pressure. *μ* is the physical viscosity, which depends on the shear rate $\dot{\gamma }=\sqrt{2D:D}$, where $D=(\nabla u+{\left(\nabla u\right)}^{T})/2$ is the strain rate tensor, and the cross-viscosity model is used:

(3) $$\mu (\dot{\gamma })=\frac{\mu_{0}}{1+{\left(\frac{\mu_{0}\dot{\gamma }}{\tau *}\right)}^{1-n}},$$

which is a 3-parameter (*μ*_0_, *n*, τ*) model.

An equation of state is also necessary to describe the relationship between density and pressure. In some previous studies of SPH [42,43], the following equations of state are commonly used:

(4) $$p(\rho )=c^{2}(\rho -\rho_{0}),$$

(5) $$p(\rho )=\frac{\rho_{0}c^{2}}{\gamma }\left[{\left(\frac{\rho }{\rho_{0}}\right)}^{\gamma }-1\right],$$

where *c* is the speed of sound, *ρ*_0_ is the initial density of the fluid, and *γ* is a constant that usually takes the value *γ* = 7. In this work, the Tait equation of state [26,44] which is especially appropriate for polymer melts is used:

(6) $$p(\rho )=B\left\{\exp\left[\frac{1}{c_{1}}\left(1-\frac{\rho_{0}}{\rho }\right)\right]-1\right\},$$

where *c*_1_ = 0.0894 for polymer melts. *B* is a very large constant parameter in the range of about 10^7^ to 10^9^ for a specific polymer melt, reflecting the compressibility of the melt.

#### 2.1.2. Improved SPH Algorithm for the Polymer Melt Injection Molding Filling Flow

In this work, an improved SPH method is applied to the macro-scale simulation. A traditional SPH discretization of the basic governing equations is shown as below [45,46]:

(7) $$\frac{d\rho_{i}}{dt}=\rho_{i}\sum_{j=1}^{N}\frac{m_{j}}{\rho_{j}}u_{ij}\cdot \nabla_{i}W_{ij},$$

(8) $$\frac{du_{i}}{dt}=-\sum_{j=1}^{N}m_{j}\left(\frac{p_{i}}{\rho_{i}{}^{2}}+\frac{p_{j}}{\rho_{j}{}^{2}}\right)\nabla_{i}W_{ij}+\sum_{j=1}^{N}m_{j}\frac{\mu_{i}+\mu_{j}}{\rho_{i}\rho_{j}}\frac{x_{ij}\cdot \nabla_{i}W_{ij}}{r_{ij}{}^{2}+{\left(0.01\;h\right)}^{2}}u_{ij}+f_{i},$$

where *m_i_*, *ρ_i_*, ***u****_i_*, *p_i_* and *μ_i_* are the mass, density, velocity, pressure and physical viscosity of the *i*-th particle, respectively, ***u****_ij_* = ***u****_i_* −***u**_j_*, ***x****_ij_* = ***x****_i_* −***x**_j_*, *r_ij_* = |***x****_ij_*|, $\nabla_{i}W_{ij}=(x_{ij}/r_{ij})\partial W_{ij}/\partial r_{ij}$, and term (0.01 *h*)^2^ in the denominator is to avoid singularity when two particles become too close, where *h* is the smooth length. The above SPH discretization is not robust and accurate enough for the high viscosity polymer melt injection molding filling flow, and certain improvements are required.

To weaken the non-physical pressure oscillation and increase the numerical accuracy and stability, a previous proposed modified low-dissipation Riemann solver and the kernel gradient correction are used, and Equations (7) and (8) are rewritten into the following forms as the main SPH algorithm in this work:

(9) $$\frac{d\rho_{i}}{dt}=\rho_{i}\sum_{j}\frac{m_{j}}{\rho_{j}}\left(u_{ij}+\frac{p_{i}-p_{j}}{\bar{\rho }\bar{c}}\frac{x_{ij}}{r_{ij}}\right)\cdot \nabla_{i}^{C}W_{ij},$$

(10) $$\frac{du_{i}}{dt}=-\sum_{j=1}^{N}\frac{m_{j}}{\rho_{i}\rho_{j}}\left(p_{i}+p_{j}-\beta_{RS}\frac{x_{ij}\cdot u_{ij}}{r_{ij}}\right)\nabla_{i}^{C}W_{ij}+\sum_{j=1}^{N}m_{j}\frac{\mu_{i}+\mu_{j}}{\rho_{i}\rho_{j}}\frac{x_{ij}\cdot \nabla_{i}^{C}W_{ij}}{r_{ij}{}^{2}+{\left(0.01\;h\right)}^{2}}u_{ij}+f_{i},$$

where $\bar{\rho }=\left(\rho_{i}+\rho_{j}\right)/2$, $\bar{c}=\left(c_{i}+c_{j}\right)/2$, $c_{i}=\sqrt{{\left(\partial p/\partial \rho \right)}_{i}}$ is the artificial speed of sound, and $\beta_{RS}=\min\left(\eta (\mu_{i}+\mu_{j})/r_{ij},\bar{\rho }\bar{c}\right)$ is the dissipation limiter based on the melt viscosity, where *η* is an adjustable parameter set as *η* = 0.5 in all the numerical examples in this work. The modified kernel gradient $\nabla_{i}^{C}W_{ij}$ is used to replace the original kernel gradient $\nabla_{i}W_{ij}$ in Equations (7) and (8), and is obtained by the following equations [47,48]:

(11) $$\nabla_{i}^{C}W_{ij}={\left(A^{s}\right)}^{-1}\cdot \nabla_{i}W_{ij},$$

(12) $$A^{s}=-\sum_{j=1}^{N}\frac{m_{j}}{\rho_{j}}x_{ij}⊗\nabla_{i}W_{ij},$$

where (***A****^s^*)^−1^ is the inverse matrix of correction matrix ***A****^s^*. To avoid singularity of ***A****^s^*, the original kernel gradient $\nabla_{i}W_{ij}$ is still used when the condition number of ***A****^s^* is larger than 10^15^.

Furthermore, the particle shifting technique [49,50] is used in this work to keep the particle distribution approximately uniform and remove the tensile instability. The particles’ shift vectors are calculated by the following two equations for inner particles and free surface particles, respectively:

(13) $$\delta x_{i}=-5h\left|u_{i}\right|\nabla C_{i}\Delta t\;\mathrm{for}\;\mathrm{inner}\;\mathrm{particles},$$

(14) $$\delta x_{i}=-5h\left|u_{i}\right|\left(I-n_{i}⊗n_{i}\right)\nabla C_{i}\Delta t\;\mathrm{for}\;\mathrm{free}\;\mathrm{surface}\;\mathrm{particles},$$

where $\nabla C_{i}=\sum_{j=1}^{N}(m_{j}/\rho_{j})\nabla_{i}W_{ij}$ is the gradient of particle concentration, Δ*t* is the time step of numerical integration, ***I*** is the 2nd-order unit tensor, and $n_{i}=A^{s}\cdot \nabla C_{i}/\left|A^{s}\cdot \nabla C_{i}\right|$ is the outer normal unit vector of free surface at the *i*-th particle, where ***A****^s^* is the same correction matrix as calculated by Equation (12).

The boundary conditions also need to be improved, and a combination of two types of virtual particles, that is, one layer of wall boundary particles and three layers of dummy boundary particles, is implemented in this work [14,29]. The velocity of a wall boundary particle is the same as the velocity boundary condition ***u****_wall_*. The pressure of a wall boundary particle is defined by a weighted average of the surrounding fluid particles’ pressures:

(15) $$p_{i}=\frac{\sum_{j=1}^{M}\frac{m_{j}}{\rho_{j}}p_{j}W_{ij}}{\sum_{j=1}^{M}\frac{m_{j}}{\rho_{j}}W_{ij}},$$

where *M* is the number of fluid particles that have interaction with the *i*-th wall boundary particle, and *j* represents their indices. For each dummy particle, its pressure is the average pressure of several wall boundary particles nearby. The velocity of this dummy boundary particle equals to the average extrapolation speed of several wall boundary particles nearby, where the extrapolation speed of a wall boundary particle ***u****_i_^ext^* is defined by the following equations:

(16) $$u_{i}^{ext}=2u_{wall}-u_{i}^{ave},\;u_{i}^{ave}=\frac{\sum_{j=1}^{M}\frac{m_{j}}{\rho_{j}}u_{j}W_{ij}}{\sum_{j=1}^{M}\frac{m_{j}}{\rho_{j}}W_{ij}},$$

where ***u****_i_^ave^* is a weighted average of the surrounding fluid particles’ velocities, which is similar to that in Equation (15). In order to avoid the contradictory velocity conditions at the intersection of the moving boundary and the fixed wall boundary, Equation (16) is only used in the normal direction of moving boundary, and ***u****_i_^ave^* is used as the tangential extrapolation speed, thus the moving boundary is equivalently frictionless.

In this work, the filling process is controlled by a uniformly moving boundary that pushes the melt forward similar to a piston, and the number of particles remains constant without new particle generation. It should be noted that the details of the above SPH algorithm improvements can be found in a previous work [30] and some will not be repeated in this article.

### 2.2. Clustered Fixed Slip-Link Model

In this work, the clustered fixed slip-link model (CFSM) [38] is applied to the micro-scale simulation, which is a coarse-grained version of the discrete slip-link model (DSM) [36,51]. Similar to the classic tube model [32,33,34,35], DSM is a single-chain mean-field model for the dense, entangled polymer chains, but includes extra information on the entanglements and chain strands between the adjacent entanglements. Empirically, a DSM simulation usually contains an ensemble of several thousand independent polymer chains. The movement of a single chain may seem stochastic, but with statistical mechanics, the statistical properties of the chain ensemble will show regularity.

In contrast with the molecular dynamics simulation, the polymer chains’ conformation is described mainly on the scale of entanglements in DSM, and the chain movement is assumed to involve two different dynamic processes: sliding dynamics (SD) and constraint dynamics (CD). SD corresponds to reallocation of Kuhn steps through entanglements with characteristic time *τ_K_*, and creation and destruction of the entanglements at the chain ends. CD is the creation and destruction of entanglements due to SD of the surrounding chains. The DSM conformation variable set of a single chain is defined as $\Omega =(Z,\{N_{i}\},\{Q_{i}\},\{\tau_{i}^{CD}\})$, where *Z* is the number of chain strands separated by *Z* − 1 entanglements, *N_i_* and ***Q****_i_* are the number of Kuhn steps and the vector, respectively, of the chain segment between the (*i* − 1)th and *i*-th entanglements, and *τ_i_^CD^* is the characteristic lifetime of the *i*-th entanglement related to CD. The conformations are described by the probability density *p*(Ω;*t*), and the equilibrium probability density *p_eq_*(Ω) is analytic and can be used to generate the initial chain ensemble configurations efficiently. The dynamics of *p*(Ω;*t*) is as follows:

(17) $$\frac{\partial }{\partial t}p(\Omega ;t)=ℒ[\Omega ;(\nabla u(t))]p(\Omega ;t),$$

where operator $ℒ[\Omega ;(\nabla u(t))]={ℒ}_{eq}[\Omega ]+{ℒ}_{flow}[\Omega ;(\nabla u(t))]$, ${ℒ}_{flow}=-\sum_{i}\left(\partial /\partial Q_{i})\cdot [{\left(\nabla u\right)}^{T}\cdot Q_{i}\right]$ is for the influence of flow, and ${ℒ}_{eq}$ keeps the equilibrium state unchanged.

In addition to the conformation variables in Ω, there are three parameters in the model: *M_K_*, the molecular weight of a Kuhn step, which is determined by chemistry and non-adjustable; *β*, which is related to the entanglement density; and *τ_K_*. By clustering a maximum number of Kuhn steps, the CFSM is derived. Mathematically CFSM is identical to DSM with fixed *β* = 1, and *M_K_*, *τ_K_* replaced by *M_c_*, *τ_c_* through the relations $M_{c}\approx 0.56(\beta +1)M_{K}$ and $\tau_{c}\approx 0.265\beta^{8/3}\tau_{K}$. The parameters of CFSM can also be obtained by matching data of small amplitude oscillatory shear (SAOS) experiments [52]. Compared to DSM, CFSM can be used for faster simulation of polydisperse polymer melt with higher average molecular weight, as is the case with most commercial injection molding polymers. Therefore, CFSM is chosen as the most suitable microscopic simulation method in this work.

### 2.3. Multiscale Simulation Solution Procedure

It should be noted that a one-way multiscale simulation is used in this work, i.e., the macro-scale simulation affects the micro-scale simulation but not vice versa. The micro-scale simulation mainly affects the macro-scale simulation by the statistical local stress to account for viscoelastic effects of the polymer melt. The influence of viscoelasticity effects on injection molding filling is generally weak, but the randomness of local stress can seriously affect the numerical stability of macro-scale simulation and therefore a one-way multiscale simulation is used. The solution procedure of the multiscale simulation is summarized as follows. First, for the macro-scale SPH method, all the fluid particles and boundary particles are generated, and the physical quantity of particles and some simulation parameters are initialized. For each SPH particle selected for the micro-scale simulation, a corresponding ensemble of several thousands of polymer chains for CFSM simulation is initialized and briefly relaxed. Then a predictor-corrector time integration scheme is used to update the physical quantity of SPH particles ***q****_i_* = (***x****_i_*, ***u****_i_*, *ρ_i_*) at the macro scale, as shown in the following equations:

(18) $$q_{i}^{n+1/2}=q_{i}^{n}+F\left(q_{i}^{n}\right)\frac{\Delta t}{2}\;\mathrm{as}\;\mathrm{predictor},$$

(19) $$q_{i}^{n+1}=q_{i}^{n}+\left(F\left(q_{i}^{n}\right)+F\left(q_{i}^{n+1/2}\right)\right)\frac{\Delta t}{2}\;\mathrm{as}\;\mathrm{corrector},$$

where ***F***(***q****_i_*) denotes to the right-hand sides of governing Equations (9) and (10), ***q****_i_^n^* and ***q****_i_^n^*^+1^ are the macro-scale physical quantity at the *n*th and (*n* + 1)th time steps, respectively. Considering the numerical stability, the time step Δ*t* should be constrained by the Courant-Friedrichs-Lewy condition, the viscous-diffusion condition and the body force condition [53]:

(20) $$\Delta t\leq \underset{\forall i}{\min}\left(0.1\frac{h}{\left|u_{i}\right|},0.125\frac{h^{2}}{\nu_{i}},\sqrt{\frac{h}{\left|f_{i}\right|}}\right),$$

where *ν_i_* = *μ_i_*/*ρ* is the kinematic viscosity, and |***f****_i_*| is the acceleration magnitude caused by external force. Considering the shear thinning polymer melt and the improvements on the SPH algorithm, the time step sizes of the macro SPH simulations are a bit larger than that strictly constrained by Equation (20), but still dramatically smaller than the micro time step size *τ_c_*. At the end of every macro time step loop, the particle shifting technique is implemented.

**Figure 1:**
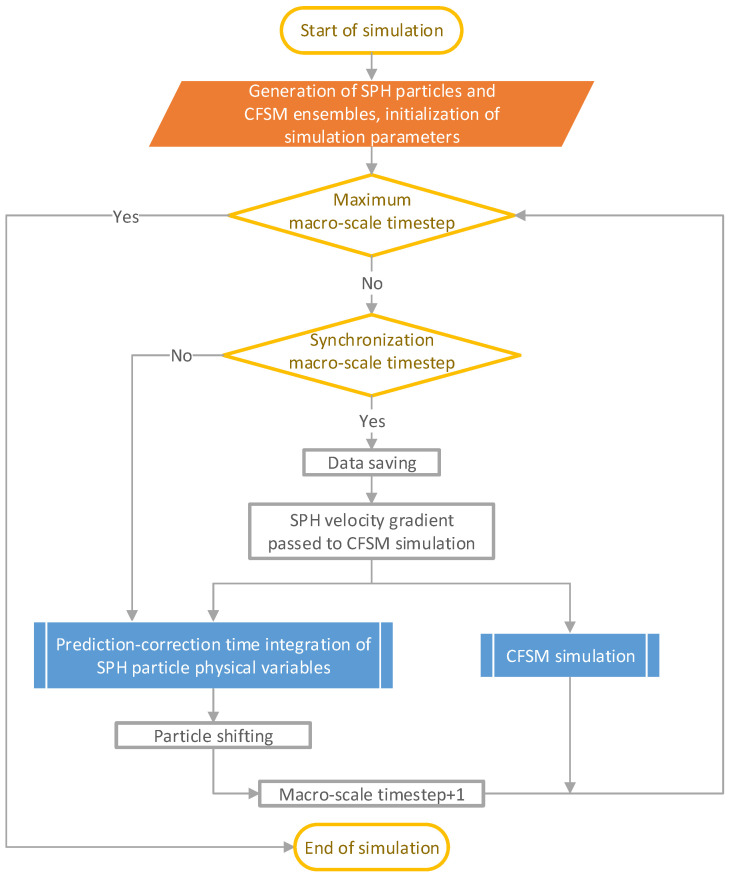
The flowchart of the multiscale simulation procedure.

Meanwhile at the micro scale, the CFSM simulation runs at a different time step, but proportional to the macro-scale time step by a rational number. Therefore, the macro-scale and micro-scale simulation times are synchronized every certain number of macro-scale time steps. At such a synchronized time, the SPH calculated macro-scale velocity gradient of each particle involved in the multiscale simulation is passed to the micro-scale CFSM simulation of the corresponding polymer chain ensemble. In addition, before the next synchronized time, the velocity gradient remains constant during the micro-scale simulation time steps. At each synchronized time, the statistical parameters calculated by micro-scale CFSM simulation are saved as well as the macro-scale SPH result data. The flowchart of the solution procedure is shown in Figure 1.

For higher computational efficiency, an open-source code called gpu_dsm [54] with CUDA acceleration is applied to the micro-scale CFSM simulation. The multiscale simulation runs on a SITONHOLY IW4210-8G workstation with 2 Intel Xeon CPUs, 128GB RAM and 8 NVIDIA GTX 1080 GPUs.

## 3. Numerical Simulation Cases

In this section, the multiscale simulation method introduced above is applied for a Poiseuille flow and two injection molding cases in two simple long 2D rectangular cavities with and without a circular obstacle, respectively. The polymer melt properties used in the numerical examples in this work are from Marlex 9006 HDPE. The macro physical properties are from the Moldflow material database. The micro properties include the molecular weight distribution (MWD) and CFSM parameters. Based on work in Ref. [52], the MWD is described by the generalized exponential (GEX) distribution:

(21) $$W(M)=\frac{b}{\Gamma \left(\frac{a+1}{b}\right)}{\left(\frac{M}{m_{p}}\right)}^{a+1}\exp\left[-{\left(\frac{M}{m_{p}}\right)}^{b}\right],$$

where *a*, *b* and *m_p_* are parameters obtained by fitting the gel permeation chromatography (GPC) experimental data [55], and Γ(*x*) is gamma function. The CFSM parameters are the same as that of PE12 sample in Ref. [52], as they depend only on temperature for the same polymer material, and the same temperature as PE12 sample is set in the simulation in this work. All the properties of Marlex 9006 HDPE are listed in Table 1. The smooth length is set as *h* = 1.5Δ*x*, where Δ*x* is the initial particle spacing. Every CFSM simulation has an ensemble of 1000 polymer chains.

### 3.1. Poiseuille Flow

**Figure 2:**
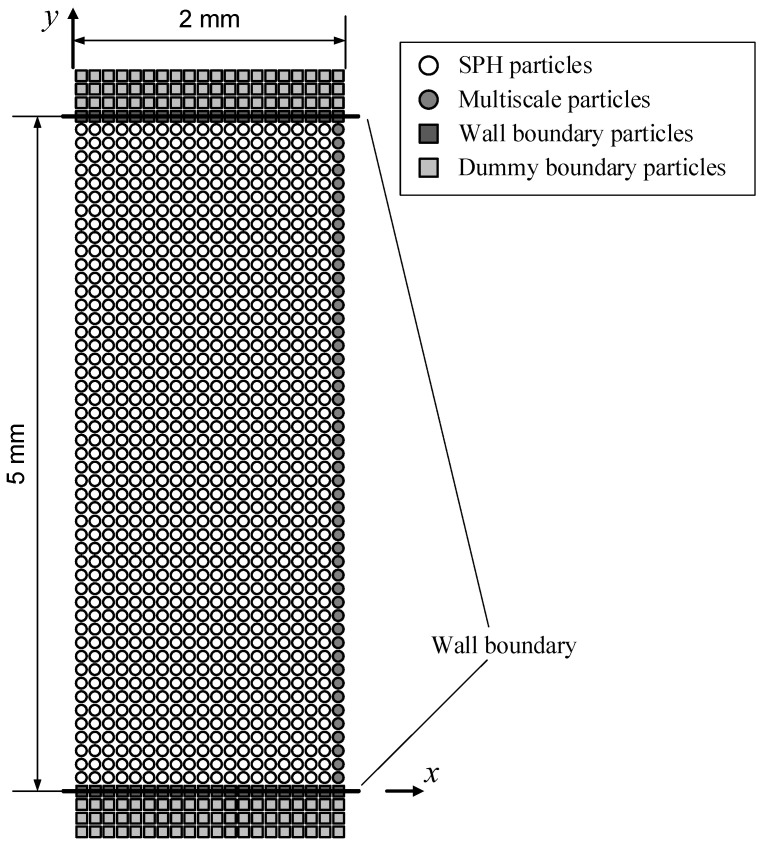
The particle arrangement of the Poiseuille flow example.

During the injection molding filling process, the velocity distribution on a section perpendicular to the fill flow direction approximates a Poiseuille flow. The particle arrangement of the Poiseuille flow example in this section is shown in Figure 2. The initial particle spacing Δ*x* = 1.0 × 10^−4^ m. Excluding the boundary particles (represented by squares), the melt particles have a total of 49 rows and 20 columns, including both the SPH particles and multiscale particles, which are all involved in the macro SPH simulation. The body acceleration for driving the Poiseuille flow ***f*** = 3.3 × 10^5^ m/s^2^ to the positive *x*-axis. Periodic boundary conditions are imposed in the *x* direction. Due to the homogeneity of Poiseuille flow in the *x* direction, only one column of particles is involved in the micro CFSM simulation (represented by grey circles as multiscale particles in Figure 2). The total simulation time is 2.25 × 10^−3^ s. To match the micro CFSM time step *τ_c_*, the macro SPH time step Δ*t* = 7.5 × 10^−9^ s = *τ_c_*/20, and the simulation runs a total of 300,000 macro time steps. The macro and micro simulations are synchronized every 200 macro (10 micro) time steps.

**Figure 3:**
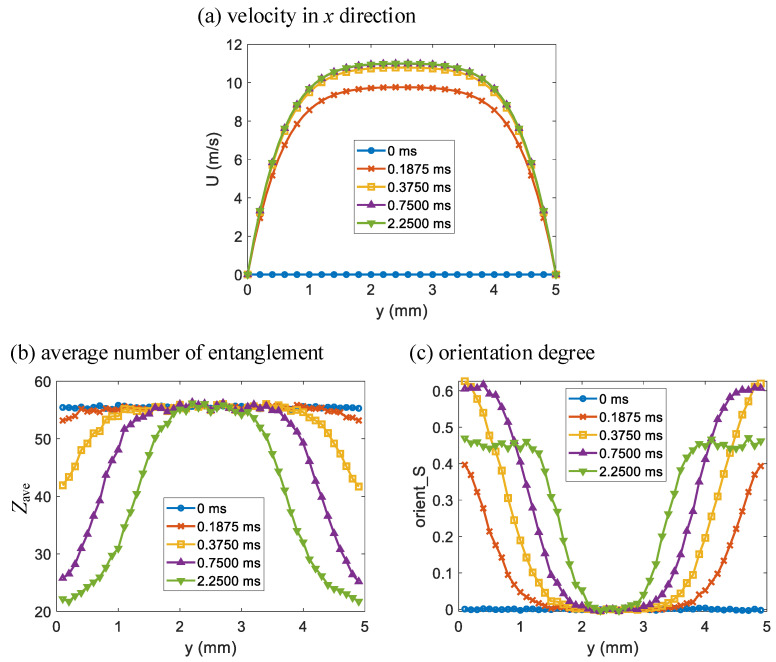
The multiscale simulation results of (**a**) velocity in *x* direction, (**b**) average number of entanglements and (**c**) orientation degree distributions in the *y* direction of Poiseuille flow at different times as indicated.

The simulated velocity in the *x* direction *U*, average number of entanglements *Z*_ave_ and orientation degree *S* distributions in the *y* direction of Poiseuille flow at times *t* = 0 s, 1.875 × 10^−4^ s, 3.75 × 10^−4^ s, 7.5 × 10^−4^ s and 2.25 × 10^−3^ s are shown in Figure 3a–c, respectively. The average number of entanglements $Z_{\mathrm{ave}}=\langle Z\rangle$, where 〈A〉 represents an average of *A* over the polymer chains in the ensemble of the corresponding CFSM simulation. The orientation degree $S=\langle 3\cos^{2}\theta -1\rangle /2$ [40], where $\cos\theta =Q_{\mathrm{ave}}\cdot Q/(\left|Q_{\mathrm{ave}}\right|\cdot \left|Q\right|)$, and $Q_{\mathrm{ave}}=\langle \mathrm{sgn}\left(Q_{x}\right)\cdot Q\rangle$, sgn is the sign function, and *Q_x_* is the *x* coordinate of the chain segment vector ***Q***. As seen in Figure 3a, *U* distribution in the *y* direction reaches steady state rapidly and has a plateau area far from the wall boundary due to the shear thinning of the polymer melt. The steady state *U* distribution in the *y* direction shows that the particle closest to the boundary has the highest shear rate, and the farther away from the boundary, the smaller the shear rate of the particle is. The particles in the plateau area of *U* distribution have insignificant shear rate. As seen in Figure 3b,c, *Z*_ave_ and *S* of the particles in the plateau area of *U* distribution have negligible change at all times. At a certain time, the particle closest to the boundary has the lowest *Z*_ave_, and the farther away from the boundary, the higher the *Z*_ave_ of the particle is, which is reasonably opposite to the trend of shear rate change due to the polymer chain disentanglement under shearing. As for the orientation degree *S*, the overall trend is the same as the shear rate change due to the polymer chain orientation under shearing, but at times 7.5 × 10^−4^ s and 2.25 × 10^−3^ s, the orientation degree *S* of several particles near the boundary are similar to each other.

**Figure 4:**
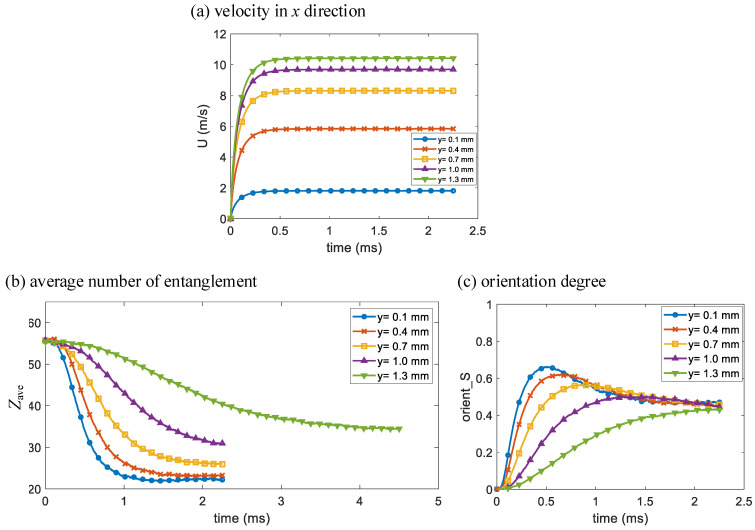
The multiscale simulation results of changes in (**a**) velocity in *x* direction, (**b**) average number of entanglements and (**c**) orientation degree over time of Poiseuille flow at different *y* coordinates as indicated.

To further analyze the simulation results, the changes in velocity in *x* direction *U*, average number of entanglements *Z*_ave_ and orientation degree *S* over time of Poiseuille flow at *y* = 0.1 mm, 0.4 mm, 0.7 mm, 1.0 mm and 1.3 mm are shown in Figure 4a–c, respectively. The *y* coordinates of the chosen particles are from closest to the lower wall boundary with the highest shear rate to near the middle of the two wall boundaries with low shear rate. As seen in Figure 4a, *U* at the chosen *y* coordinates basically reaches steady state at time *t* = 5 × 10^−4^ s, thus the states of the particles are close to pure shear at different shear rates. As seen in Figure 4b, *Z*_ave_ of a certain particle decreases over time under shearing and decreases faster with higher shear rates. However, *Z*_ave_ eventually decreases to a minimum and remains almost constant, which indicates the equilibrium state of shear disentanglement. The simulation of *Z*_ave_ change over time at *y* = 1.3 mm in Figure 4b is specifically extended to capture its steady state. *Z*_ave_ of a particle with lower shear rate decreases to a higher minimum. As seen in Figure 4c, *S* of a certain particle first increases over time under shearing and increases faster with higher shear rates. For a particle with low shear rate, its *S* eventually increases to a maximum remains almost constant. While for a particle with high shear rate, its *S* first increases to a maximum, and then decreases to a certain value and remains almost constant, which indicates relaxation in shear orientation. *S* of a particle with higher shear rate increases to a higher maximum, but *S* of the chosen particles all decrease to a similar value.

The above Poiseuille flow occurs in the injection molding filling of most thin-walled products. The micro *Z*_ave_ and *S* results indicate that polymer melt close to the cavity wall is well disentangled and oriented while melt in the center is not. This variance causes the skin-core structure along the thickness direction in a thin-walled injection molded product.

### 3.2. Injection Molding Filling in a Simple Long Rectangular Cavity

**Figure 5:**
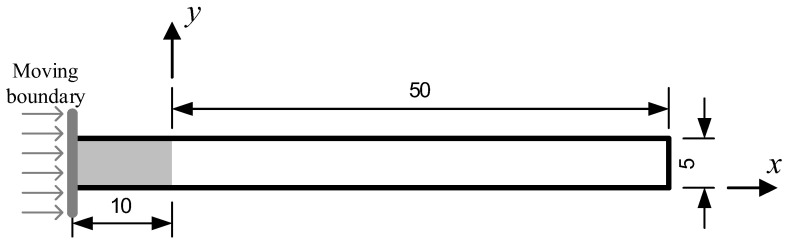
The sketch of the simple long rectangular cavity (unit: mm).

The rectangular cavity is one of the simplest cavities. It can be regarded as the simplified 2D model of a plate or an injection runner. The sketch of the simple long rectangular cavity in this section is shown in Figure 5. The black lines indicate the fixed boundaries as the cavity inner walls. The grey line indicates the moving boundary as the injection piston, and its velocity is constant at 10 m/s along the positive *x*-axis. The grey area is initially filled with fluid particles as the polymer melt. The initial particle spacing Δ*x* = 1.25 × 10^−4^ m, hence there are a total of 39 rows and 80 columns of fluid particles, which are all involved in both macro SPH and micro CFSM simulations. It takes 0.005 s for the melt to fully contact the right cavity wall as estimated. To match the micro CFSM time step *τ_c_*, the macro SPH time step Δ*t* = 1.5 × 10^−8^ s = *τ_c_*/10, and the simulation requires a total of 333,600 macro time steps. The macro and micro simulations are synchronized every 300 macro (30 micro) time steps.

**Figure 6:**
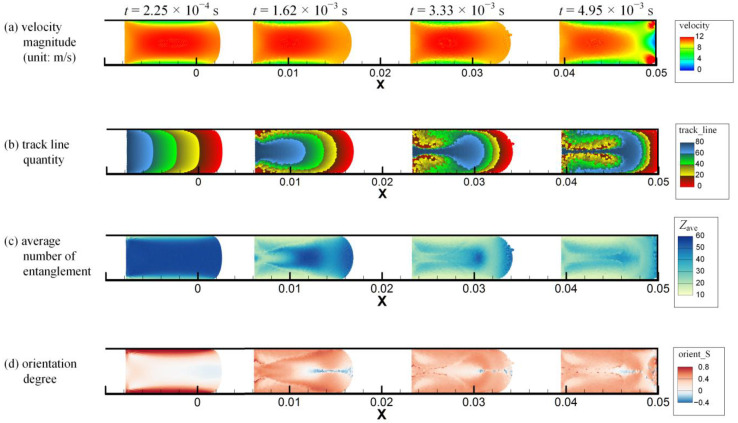
The multiscale simulation results of (**a**) velocity magnitude, (**b**) track line quantity, (**c**) average number of entanglements and (**d**) orientation degree distributions of injection molding in the simple long rectangular cavity at different times as indicated.

The simulated velocity magnitude, track line quantity, average number of entanglements *Z*_ave_ and orientation degree *S* distributions of injection molding in the simple long rectangular cavity at times *t* = 2.25 × 10^−4^ s, 1.62 × 10^−3^ s, 3.33 × 10^−3^ s and 4.95 × 10^−3^ s are shown in Figure 6a–d, respectively. The animations corresponding to the above results can be found in Supplementary Material Figures S1–S4. At the beginning of the injection, *Z*_ave_ and *S* near the cavity wall rapidly decrease and increase, respectively, which is similar to the previous Poiseuille flow example. During the injection process, the velocity distribution seems to remain stable in the polymer melt most of the time. The track line quantity of the rightmost column of melt particles at *t* = 0 s is defined as zero. Then the track line quantity of each column of particles is increased by one from right to left and remains constant for each particle during the simulation. As seen in the track line quantity result, melt tumbling occurs near the melt front (free surface) and the injection piston (moving boundary), and the micro *Z*_ave_ and *S* results are also affected. *Z*_ave_ and *S* in the center area of the melt changes little at the beginning of the injection. As the melt near the cavity wall gradually tumbles to the center area, it experiences shearing near the cavity wall and the piston. Hence the melt with lower *Z*_ave_ and higher *S* gradually occupies the center area.

**Figure 7:**
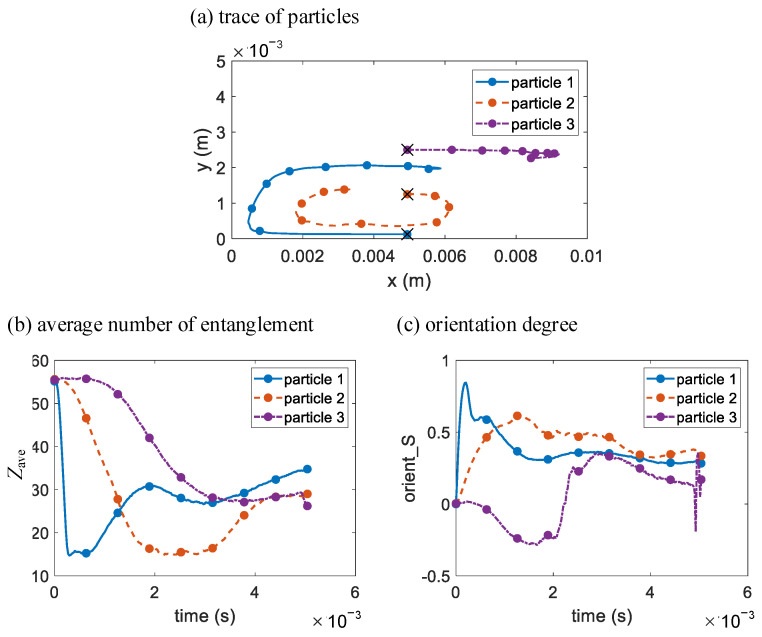
The multiscale simulation results of (**a**) particle traces and the changes in (**b**) average number of entanglements and (**c**) orientation degree over time of 3 chosen melt particles in the simple long rectangular cavity.

For a more detailed view of the simulation results, 3 melt particles are chosen and their traces with the injection piston (*x* = 0 in Figure 7a) as the reference frame are shown in Figure 7a, and their changes in average number of entanglements *Z*_ave_ and orientation degree *S* over time are shown in Figure 7b,c, respectively. At time *t* = 0 s, all 3 chosen particles are at *x* = 0.005 m as marked by the 3 crosses in Figure 7a, and particle 1 abuts against the inner cavity wall, particle 3 is at the center of the melt, and particle 2 is at the midpoint of the above two. During the injection, particle 1 remains against the inner wall and is subjected to intense shearing until it moves close to the piston, meanwhile its *Z*_ave_ is reduced to a minimum, and its *S* increases to a maximum and then decreases due to relaxation. Then particle 1 gradually tumbles to the middle of the injection piston and moves away from it, the shearing should be weak near the frictionless piston, but two flows converge near the middle of the piston, which causes *Z*_ave_ decreases slightly after a rapid increase while *S* does the opposite. After that, particle 1 gradually moves close to the melt center, and the shearing is weak away from the inner wall, *Z*_ave_ increases slowly and *S* remains at a low value with slight drop. As for particle 2, as it tumbles close to the inner wall, it is subjected to shearing weaker than particle 1, hence it has a slower rate of *Z*_ave_ decrease and *S* increase and relaxation. Then as particle 2 moves towards the piston, its *Z*_ave_ and *S* almost remain constant. Next, as particle 2 tumbles away from the inner wall, it is subjected to weakened shearing, and its *Z*_ave_ increases a bit but remains lower than that of particle 1, its *S* decreases but remains slightly higher than that of particle 1. Particle 3 stays in the middle of the *y* direction in the melt, and slowly move to the melt front because of the fountain flow effect. There is tensile force near the melt front, hence *Z*_ave_ of particle 3 decreases due to stretching orientation, and *S* of particle 3 becomes negative, indicating the tendency of biaxial orientation of polymer chains. As particle 3 moves closer to the melt front, it has a greater degree of stretch orientation, and its *Z*_ave_ decreases more slowly and tends to be constant, and its *S* become positive, indicating the polymer chains being uniaxially oriented again. At last, after the melt front contacts the right cavity wall, particle 3 encounters transient pressure and shear, and its *Z*_ave_ suddenly decreases, and its *S* has a big fluctuation.

In contrast with the Poiseuille flow example, the above flow patterns mainly occur near the melt front and in the barrel of an injection molding machine. The melt near the melt front is also disentangled and oriented but less than that close to the cavity wall. When the injection molding filling stage is over, the stretching-oriented melt front may cause shrinkage and warpage at the end edge of a product. As indicated above, the sheared melt near the injection piston can tumble to the middle, and this part of melt in the barrel may enter the runner system or even the product cavity through the injection gate. To prevent this part of the melt from entering the product cavity, the runner system should be long enough and have corners.

### 3.3. Injection Molding Filling in a Rectangular Cavity with a Circular Obstacle

**Figure 8:**
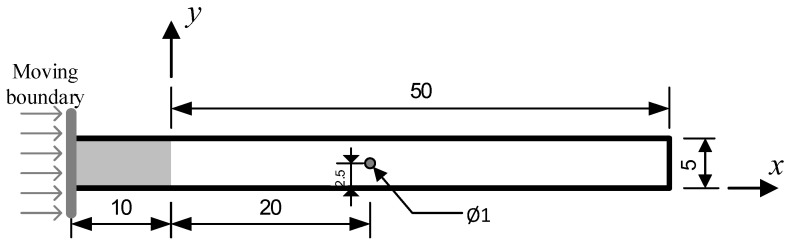
The sketch of the rectangular cavity with a circular obstacle (unit: mm).

In order to further analyze the evolution of polymer chain conformation in injection molding, on the basis of the simple rectangular cavity in Section 3.2, a small circular obstacle is added to the center of the cavity, and the multiscale simulation results in this section can be compared with that in Section 3.2. The sketch of the rectangular cavity with a circular obstacle is shown in Figure 8. The circular obstacle is regarded as fixed wall boundary. The velocity of the injection piston, the initial particle spacing, and the simulation macro and micro time step and synchronization are just the same as in Section 3.2.

**Figure 9:**
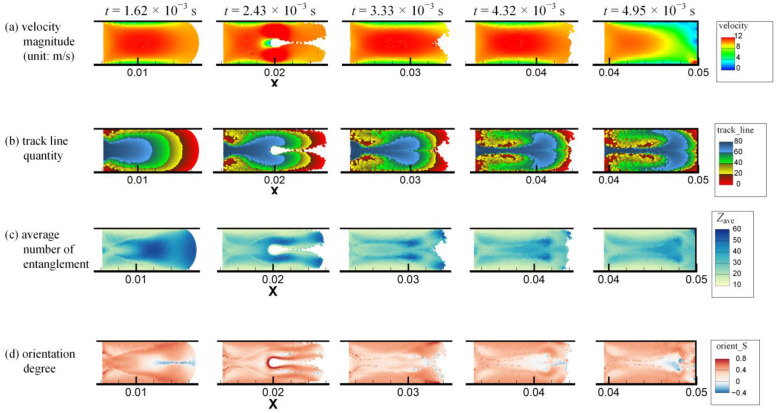
The multiscale simulation results of (**a**) velocity magnitude, (**b**) track line quantity, (**c**) average number of entanglements and (**d**) orientation degree distributions of injection molding in the rectangular cavity with circular obstacle at different times as indicated.

The simulated velocity magnitude, track line quantity, average number of entanglements *Z*_ave_ and orientation degree *S* distributions of injection molding in the rectangular cavity with circular obstacle at times *t* = 1.62 × 10^−3^ s, 2.43 × 10^−3^ s, 3.33 × 10^−3^ s, 4.32 × 10^−3^ s and 4.95 × 10^−3^ s are shown in Figure 9a–d, respectively. The animations corresponding to the above results can be found in Supplementary Material Figures S5–S8. The results at time *t* = 1.62 × 10^−3^ s are the same as in Figure 6, as the melt is not in contact with the obstacle at that time. After the melt contacts with the obstacle, the melt is split in half, and then rapidly merged back together. After the melt passes the obstacle, the shape of the melt front changes significantly, and tends to change back to its original shape as the injection progresses. Furthermore, more melt near the melt front (indicated by red color in Figure 9b) tumbles to the cavity wall due to the fountain flow effect compared with the result in Section 3.2 without obstacle in Figure 6. When the melt is passing the obstacle, the velocity of the melt on the upper and lower sides of the obstacle increases. However, due to the characteristic of Poiseuille flow, strong shearing occurs only close to the cavity walls and the obstacle, thus two banded areas on the upper and lower sides of the obstacle and symmetrical about the obstacle with higher *Z*_ave_ and lower *S* can be seen in Figure 9c,d, respectively, at time *t* = 3.33 × 10^−3^ s. Then traces of the two banded areas are still evident in the *Z*_ave_ result in Figure 9c but become blurred in the *S* result in Figure 9d at time *t* = 4.32 × 10^−3^ s due to relaxation.

**Figure 10:**
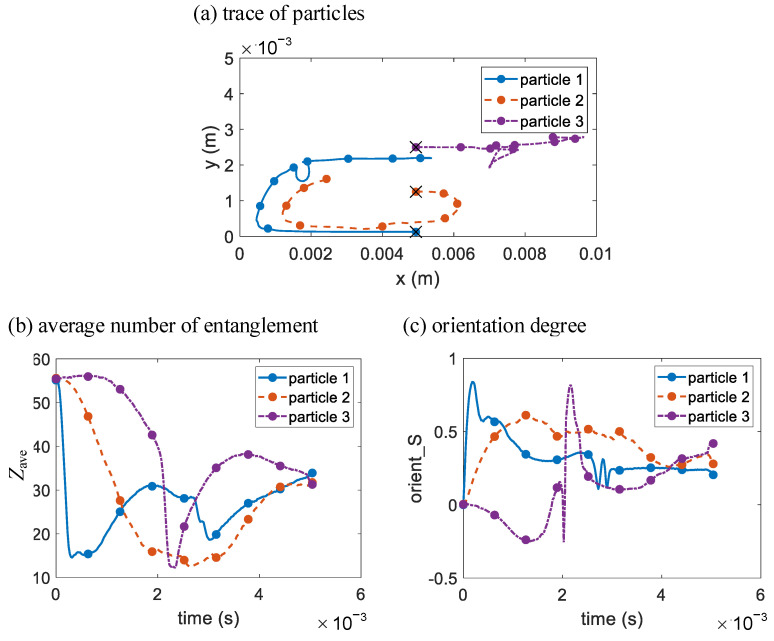
The multiscale simulation results of (**a**) particle traces and the changes in (**b**) average number of entanglements and (**c**) orientation degree over time of 3 chosen melt particles in the rectangular cavity with circular obstacle.

For comparison, the multiscale simulation results of the same 3 chosen particles as in Figure 7 are plotted. Their traces with the injection piston (*x* = 0 in Figure 10a) as the reference frame are shown in Figure 10a, and their changes in average number of entanglements *Z*_ave_ and orientation degree *S* over time are shown in Figure 10b,c, respectively. Before the melt contacts with the obstacle, the results are the same as in Section 3.2. As seen in Figure 10a, traces of all the 3 chosen particles are slightly affected by the obstacle. Particle 1 is closest to the obstacle when it just tumbles to the middle of the injection piston and is moving away from it. As particle 1 and the obstacle are still a certain distance away, the flow stress particle 1 is subjected to are not very large, and *Z*_ave_ of particle 1 decreases a bit and then continuously increases when particle 1 moves away from the obstacle. Meanwhile *S* of particle 1 has a small fluctuation, which indicates that the flow stress particle 1 is subjected to is normal stress dominated with little shearing. Compared to results in Figure 7, particle 2 is pushed closer to the inner wall when the melt passes the obstacle, and then tumbles more away from the inner wall, hence *Z*_ave_ decreases a bit more when the melt passes the obstacle and then increases more, while *S* has the opposite tendency. As for particle 3, as it stays in the middle of the *y* direction in the melt, particle 3 hits the obstacle head-on, and then moves close to the obstacle and eventually moves away from the obstacle with one half of the split melt, and then moves back to the middle as the melt rapidly merged back together. When particle 3 moves close to the obstacle, particle 3 is subjected to intense shearing, and *Z*_ave_ rapidly decreases to minimum. *S* of particle 3 first instantaneously decreases to negative due to transient pressure when particle 3 hits the obstacle, and then rapidly increases to maximum. After particle 3 moves away from the obstacle, *Z*_ave_ rapidly increases, and *S* rapidly decreases. Due to the influence of the obstacle on the fountain flow at the melt front, particle 3 moves closer to the melt front than the result in Figure 7a, and is subjected to more intense stretching, which causes *Z*_ave_ decrease and *S* increase. At last, after the melt front contacts the right cavity wall, *Z*_ave_ of particle 3 further decreases, while *S* does the opposite.

Obstacles are common in injection molds and correspond to features such as holes in injection molded parts. As indicated above, melt close to the obstacle is subjected to intense shearing and briefly split in half after passing through the obstacle. If the obstacle is away from the end edge of the product cavity, the influence of obstacle on the melt front will gradually weaken. However, weld line may occur in the product where melt is briefly split in half after passing through the obstacle, and cause appearance and performance issues.

## 4. Conclusions

In this work, a multiscale simulation of injection molding filling flow is implemented in various examples. The multiscale simulation approach is established by combining an improved SPH method and CFSM. A modified low-dissipation Riemann solver with some other improvements is used in the SPH method to achieve the macro injection molding filling simulation. The GPC experiment is carried out to obtain the GEX distribution parameters used in the CFSM. For each simulation example, the macro velocity results, and the micro average number of entanglements *Z*_ave_ and orientation degree *S* results of multiscale simulation are demonstrated and discussed. In the two injection molding filling examples, the above results together with the tracking line quantity results of the two examples are compared. Some changing trends of *Z*_ave_ and *S* are obtained. In the Poiseuille flow example, distribution of velocity in the *x* direction reaches steady state rapidly with high-shear steep area near the wall boundary and a low-shear plateau area far from the wall boundary. *Z*_ave_ and *S* change negligibly in the plateau area. At a higher shear rate, *Z*_ave_ decreases faster and eventually reaches equilibrium at a smaller minimum, and *S* increases faster and reaches a higher maximum, but then decreases when the shear rate is too high, and eventually equilibrates to a similar value regardless of the shear rate. The variance in the plateau area and near the wall boundary causes the skin-core structure along the thickness direction in a thin-walled injection molded product. In the example of injection molding filling in a simple long rectangular cavity, melt tumbling occurs near the melt front and the injection piston. *Z*_ave_ and *S* in the melt center area changes little at the beginning of the injection. As the melt near the cavity wall gradually tumbles to the center area, the sheared melt with lower *Z*_ave_ and higher *S* gradually occupies the center area. The runner system should be long enough with corners to prevent the above sheared melt from entering the product cavity. The stretching-oriented melt front may cause shrinkage and warpage at the end edge of a product. In the example of injection molding filling in a rectangular cavity with a circular obstacle, the melt is split in half after contact with the obstacle, and then rapidly merged back together. After the melt passes the obstacle, the shape of the melt front tends to change back to its original shape as the injection progresses, and more melt near the melt front tumbles to the cavity wall due to the fountain flow effect compared with the example of injection filling in a simple long rectangular cavity without obstacle. Furthermore, multiscale simulation results of 3 chosen particles in the two injection molding filling examples are compared. The comparison indicates that the obstacle has little effect on the overall trace of particles, but obviously disturbs the changing trends of *Z*_ave_ and *S*. The closer the particle is to the obstacle, the more the result is affected. Weld line may occur in the product where melt is briefly split in half after passing through the obstacle, and the influence of obstacle on the melt front will gradually weaken when the melt front moves far away.

In this work, all the multiscale numerical examples are 2D isothermal problems. In the future work, the macro SPH method can be extended to 3D problems and incorporate heat conduction and phase transitions to simulate injection molding packing and cooling processes after filling stage. More rheological experiments are needed to extend the CFSM parameter database to various polymer melts at different temperatures. The micro statistical local stress can be transferred back to the macro simulation to enable a complete multiscale simulation of viscoelastic polymer melt.

## Figures and Tables

**Table 1:** The properties of Marlex 9006 HDPE.

	Parameter	Value
SPH	Initial melt density, *ρ*_0_ (kg/m^3^)	742.93
	Melt temperature, *T* (K)	463.15
	Zero-shear viscosity, *μ*_0_ (Pa·s)	1254.18
	Critical stress level at the transition to shear thinning, τ* (Pa)	192,149
	Power law index in the high shear rate regime, *n*	0.2411
	Compressibility parameter of the Tait model, *B* (Pa)	7.9344 × 10^7^
GEX	Shape parameter, *a*	1.39
	Shape parameter, *b*	0.26
	Localization parameter, *m_p_* (g/mol)	20.54
CFSM	Molecular weight of a Kuhn step cluster, *M_c_* (g/mol)	1089.12
	Characteristic time for a Kuhn step cluster, *τ_c_* (s)	1.5 × 10^−7^

## Data Availability

Not applicable.

## Supplementary Materials

The following supporting information can be downloaded at: https://www.mdpi.com/article/10.3390/polym14204334/s1, Figure S1: Animation of velocity magnitude distribution over time of injection molding in the simple long rectangular cavity; Figure S2: Animation of track line quantity distribution over time of injection molding in the simple long rectangular cavity; Figure S3: Animation of average entanglement number distribution over time of injection molding in the simple long rectangular cavity; Figure S4: Animation of orientation degree distribution over time of injection molding in the simple long rectangular cavity; Figure S5: Animation of velocity magnitude distribution over time of injection molding in the rectangular cavity with circular obstacle; Figure S6: Animation of track line quantity distribution over time of injection molding in the rectangular cavity with circular obstacle; Figure S7: Animation of average entanglement number distribution over time of injection molding in the rectangular cavity with circular obstacle; Figure S8: Animation of orientation degree distribution over time of injection molding in the rectangular cavity with circular obstacle.
