# Supplementary figures and images for: A Multiscale Simulation of Polymer Melt Injection Molding Filling Flow Using SPH Method with Slip-Link Model
Source: Polymers (Basel). 2022 Oct 14;14(20):4334. doi: 10.3390/polym14204334 (PMC9612108; doi:10.3390/polym14204334)

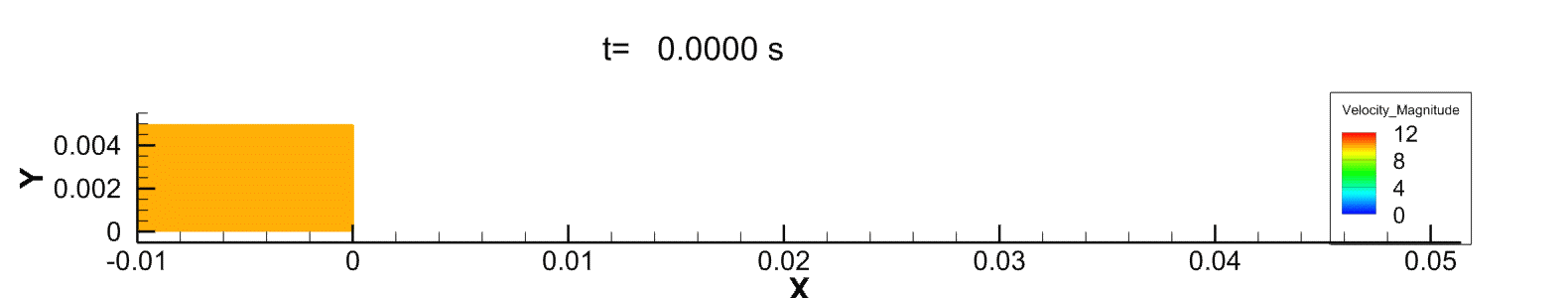

Supplement: Supplementary file 1 [file polymers-14-04334-s001.zip › polymers-1917355-supplementary/gif (supplementary material)/Figure S1_injection_rect_v_abs.gif]

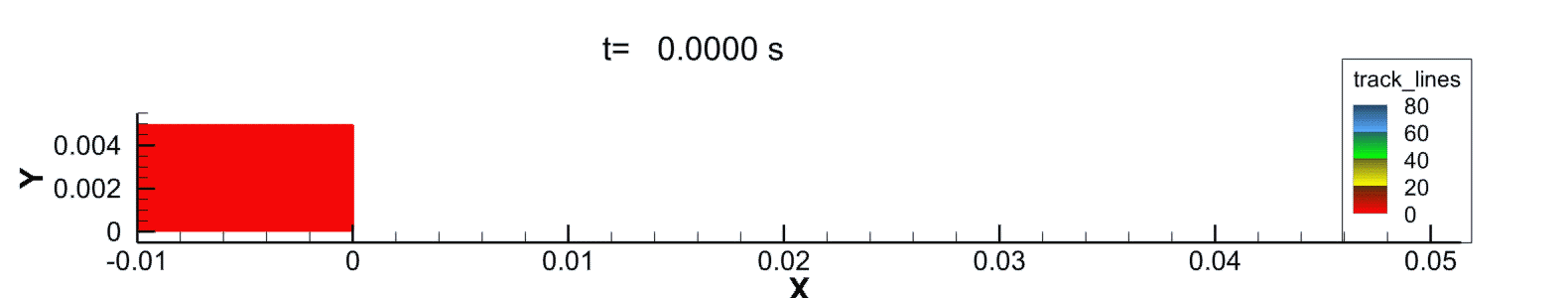

Supplement: Supplementary file 1 [file polymers-14-04334-s001.zip › polymers-1917355-supplementary/gif (supplementary material)/Figure S2_injection_rect_track_line.gif]

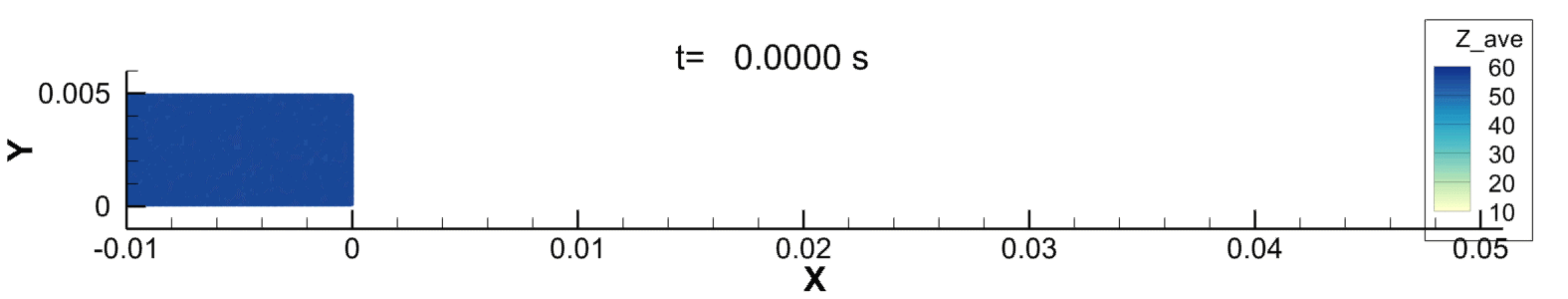

Supplement: Supplementary file 1 [file polymers-14-04334-s001.zip › polymers-1917355-supplementary/gif (supplementary material)/Figure S3_injection_rect_Z_ave.gif]

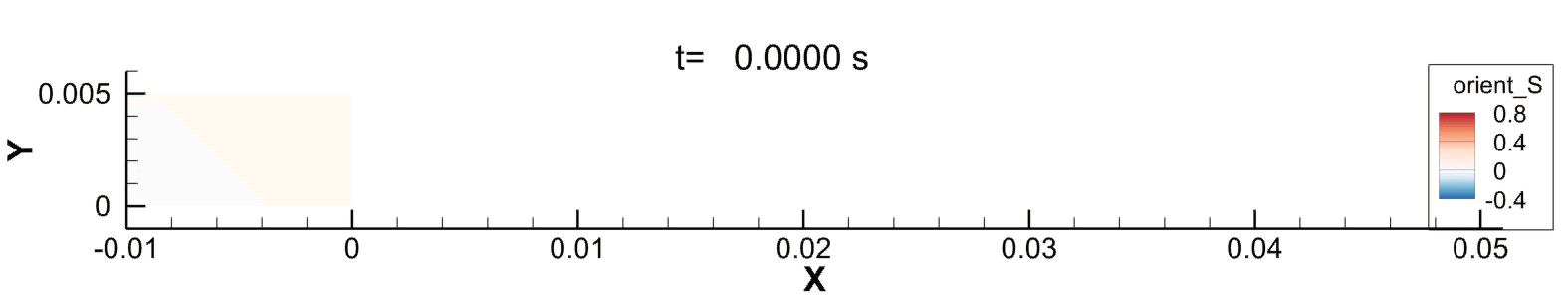

Supplement: Supplementary file 1 [file polymers-14-04334-s001.zip › polymers-1917355-supplementary/gif (supplementary material)/Figure S4_injection_rect_orient_S.gif]

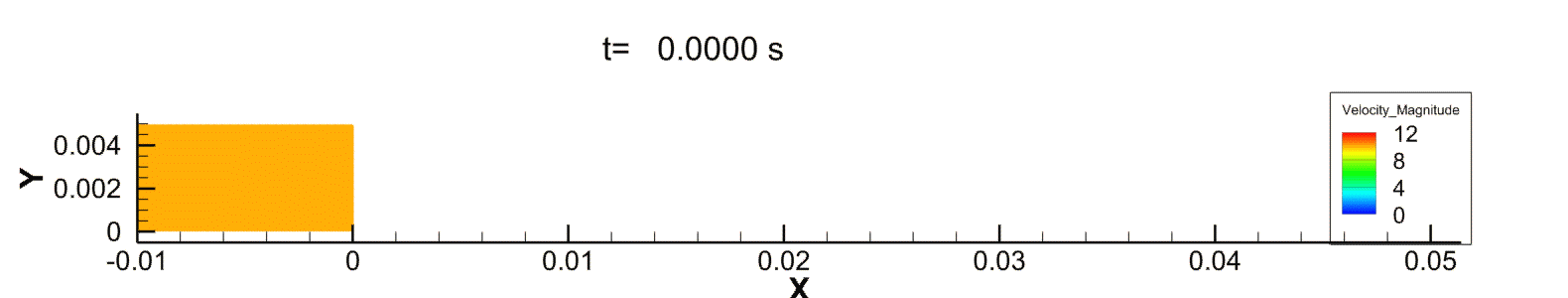

Supplement: Supplementary file 1 [file polymers-14-04334-s001.zip › polymers-1917355-supplementary/gif (supplementary material)/Figure S5_injection_obstacle_v_abs.gif]

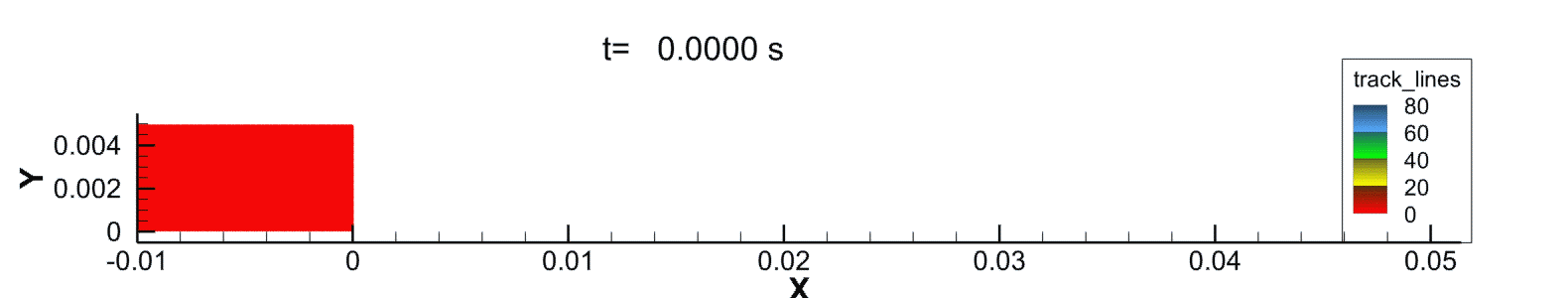

Supplement: Supplementary file 1 [file polymers-14-04334-s001.zip › polymers-1917355-supplementary/gif (supplementary material)/Figure S6_injection_obstacle_track_line.gif]

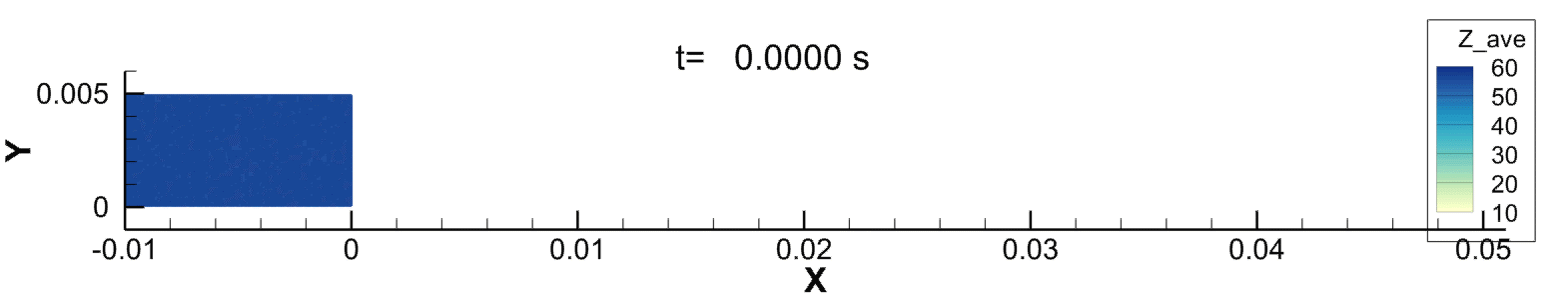

Supplement: Supplementary file 1 [file polymers-14-04334-s001.zip › polymers-1917355-supplementary/gif (supplementary material)/Figure S7_injection_obstacle_Z_ave.gif]

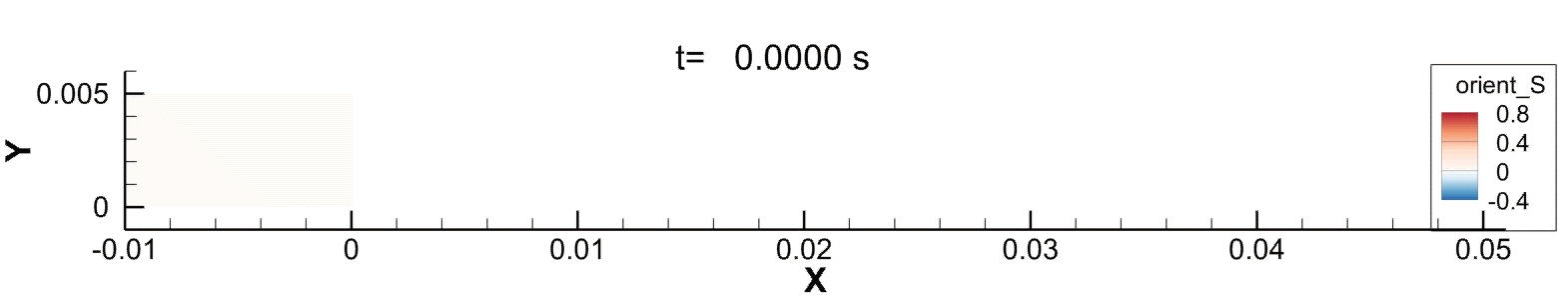

Supplement: Supplementary file 1 [file polymers-14-04334-s001.zip › polymers-1917355-supplementary/gif (supplementary material)/Figure S8_injection_obstacle_orient_S.gif]
